# Periodontitis and gestational diabetes mellitus: a systematic review and meta-analysis of observational studies

**DOI:** 10.1186/s12884-016-1145-z

**Published:** 2016-11-08

**Authors:** Samuel A. Abariga, Brian W. Whitcomb

**Affiliations:** Division of Biostatistics and Epidemiology, School of Public Health and Health Sciences, University of Massachusetts, 415 Arnold House, 715 North Pleasant Street, Amherst, MA 01003 USA

**Keywords:** Meta-analysis, Gestational diabetes, Inflammation, Periodontal disease, Pregnancy

## Abstract

**Background:**

Gestational diabetes mellitus (GDM) is glucose intolerance with first onset during pregnancy and is associated with serious maternal and fetal complications. The etiology of GDM is not well understood, but systemic inflammation effects on insulin signaling and glucose metabolism is suspected. Periodontal disease is a chronic inflammatory condition that induces local and host immune responses and has been evaluated for a potential role in development of GDM. Results from studies evaluating the association between periodontitis and GDM are mixed. We performed a systematic review and meta-analysis to summarize available data regarding the association between periodontitis and GDM.

**Methods:**

Twelve electronic databases were searched for observational studies of the association between periodontitis and GDM through March 2016. Eligible studies were assessed for quality and heterogeneity. Random effects models were used to estimate summary measures of association.

**Results:**

We identified 44 articles from 115 potentially relevant reports of which 10 studies met our eligibility criteria. Clinical diagnostic criteria for periodontitis and GDM varied widely among studies, and moderate heterogeneity was observed. Random effects meta-analysis of all included studies with a total of 5724 participants including 624 cases, showed that periodontitis is associated with an increased risk of GDM by 66 %, (OR = 1.66, 95 % CI: 1.17 to 2.36; *p* < 0.05), I^2^ = 50.5 %. Similar results were seen in sub-analysis restricted to data from methodologically high quality case–control studies including 1176 participants including 380 cases, (OR = 1.85, 95 % CI: 1.03 to 3.32); *p* < 0.05), I^2^ = 68.4 %. Meta-analysis of studies that adjusted for potential confounders estimated more than 2-fold increased odds of GDM among women with periodontitis (aOR = 2.08, 95 % CI: 1.21 to 3.58, *p* = 0.009, I^2^ = 36.9 %).

**Conclusion:**

Meta-analysis suggests that periodontitis is associated with a statistically significant increased risk for GDM compared to women without periodontitis. Robust prospective study designs and uniform definition for periodontitis and GDM definitions are urgently needed to substantiate these findings.

## Background

Gestational diabetes mellitus (GDM) is glucose intolerance with first onset during pregnancy [1]. GDM is believed to affect approximately 15 % of all pregnant women worldwide [2]. The prevalence of GDM in the United States between 2007 and 2010 was estimated to be 9.2 % [3]. Patients at risk of GDM are also at increased risk for fetal complications, such as fetal macrosomia, shoulder dystocia, neonatal hypoglycemia and maternal complications such as preeclampsia [4]. GDM is also associated with a 7-fold increased risk for developing type 2 diabetes mellitus [5]. The etiology of GDM is not well understood, but recognized risk factors for GDM include obesity [6], family and previous history of GDM, advanced maternal age and polycystic ovarian syndrome [7], as well as cigarette smoking and non-white race [8–10]. Elevated leukocytes and C-reactive protein in systemic circulation of women with GDM have been reported. Chronic systematic inflammation, which is known to impact insulin resistance, may play a role in the development of GDM [11–13].

Periodontitis is a chronic inflammatory condition that affects supporting structures of the teeth and is induced by the presence of a microbial biofilm on the surface of teeth [14]. Periodontitis affects approximately 65 million (47 %) US adults 30 years and older, [15] and about 6 % of women in child bearing age [16]. Prior research has linked periodontitis with risk of adverse health outcomes including diabetes [17]. It is thought that bacteria and bacterial products such as lipopolysaccharide from the sub gingival plaque result in the release of pro-inflammatory cytokines (tumor necrosis factor-alpha, interleukin-1beta, interleukin-6, interleukin-8, and C-reactive protein) from the inflamed periodontal tissue [18], which enter circulation and interfere with insulin signaling and causing insulin antagonism and pancreatic β-cell destruction [19, 20]. The sustained elevation of these cytokines is believed to interfere with carbohydrate metabolism and glucose tolerance [19].

Epidemiologic studies of periodontal disease among pregnant women have observed associations with low birth weight and preterm birth [21–23]. Results from studies investigating the link between periodontitis and GDM are mixed [24–27], though relations with risk of type 2 diabetes, poor glycemic control, and diabetes complications are supported [28]. Results from previous reviews on the association between periodontitis and increased adverse birth and pregnancy outcomes either did not address the effect of periodontitis on GDM [29] or were inconclusive, potentially related to the limited number of available studies and related issues with statistical power [30]. Establishing a link between periodontitis as a risk factor for gestational diabetes may provide new public health intervention strategies for the prevention of gestational diabetes and its adverse effects on pregnancy outcome.

In this review, we evaluate the association between periodontitis and GDM by systematically appraising studies on periodontitis and GDM and perform a quantitative assessment of the association of periodontitis with risk of GDM.

## Methods

We followed a standard protocol based on the Preferred Reporting Items for Systematic Reviews and Meta-Analyses statement [31].

### Data sources and search strategy

We performed a comprehensive search of the following databases; Medline; the Cochrane database of controlled trials and systematic reviews; PsychINFO; CAB; NHS Economic Evaluation Database; Health Star; SCOPUS; EMBASE; CINAHL; Google Scholar; Global Health and Health Watch; through April 2015, and updated our search in March 2016. References and bibliographic lists of selected studies were also searched. We used search terms including; “Periodontitis”, “Periodontal disease”, “Gum disease”, “Oral health”, “Gingivitis”, “Dental plague”, “Periodontal pathology”, “Diabetes mellitus”, “Gestational diabetes mellitus”, “Pregnancy induced diabetes”, “Hyperglycemia”, “High blood glucose levels”; their exploded Mesh terms related key words were used as well.

### Study selection

Studies of the relationship between periodontitis and risk of GDM were eligible for selection if they: were published in English language; used analytical observational study designs (*i.e*., cohort, case–control, cross-sectional); were conducted among pregnant women; clearly defined GDM by either the one or two-step approach [32–35]: defined periodontitis using at least one of several clinical definitions according to the International Workshop for the Classification of Periodontal Disease [36] or by self-report; and provided measures of association such as relative risk (RR), odds ratio (OR) or hazard ratio (HR) or data to allow for computation of summary measures. Where multiple measures were used to define periodontal disease in a single study, we chose clinical attachment loss (CAL) over probing depth (PD), which was chosen over radiographs to define periodontitis [36]. Studies were excluded if: GDM was assessed as the exposure and periodontitis as the outcome; they were reviews or case reports; and, if they lacked information for calculation of risk estimates. In instances where more than one publication appeared from the same study, data from the most inclusive report were used.

### Data extraction

We screened abstracts of electronic citations and retrieved full articles of studies that met all predetermined criteria for detailed review. Two authors (SAA and BWW) independently assessed the characteristics of included studies and abstracted information on authors, country, publication year, study design, setting, study population, mean age/age range, exposure and outcome definitions, data including counts and/or effect estimates, variables included in adjusted models, as well as authors’ main conclusions.

### Quality assessment

We assessed quality of included case–control and cohort studies using the Newcastle Ottawa Scale (NOS) for nonrandomized trials based on study group selection, their comparability and the ascertainment of either the exposure or outcome of interest for case–control or cohort studies respectively [37]. On the basis of the NOS, we scored each study as high quality for studies receiving at least 8 stars; medium quality for those awarded seven stars, or low quality if studies had fewer than seven stars. We did not assess the quality of cross sectional studies.

### Data synthesis and statistical analysis

Meta-analysis was performed using DerSimonian–Laird random-effects models in order to address variation across studies related to measurement of periodontitis and GDM as well as study designs [38]. To assess the overall risk estimate, we pooled crude risk estimates from all studies; in addition, analyses were conducted separately, based on study design. Additional analyses were conducted using confounder-adjusted estimates from studies with this information available, and a subgroup analysis restricted to studies with high methodologic quality that provided information on adjusted measures was performed as well. Two tailed *p*-values were used, and *P <* 0.05 was used to define statistical significance. Heterogeneity was assessed using the *I* ^*2*^ statistic and *Q* test [39]. Heterogeneity was considered to be significant at *P* < 0.1. We assessed publication bias by visual inspection of funnel plots of the log odds ratio against its standard error and assessed the degree of funnel plot asymmetry using Egger’s unweighted regression asymmetry test [40]. All analyses were conducted using Stata (version 12.1; StataCorp, College Station, TX) [41].

## Results

### Study selection and study characteristics

**Fig. 1:**
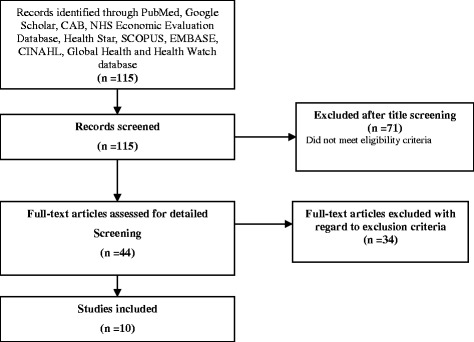
Summary of search yield and selection

Our comprehensive literature search yielded a total of 115 abstracts, from which 44 articles were retrieved for full text review after removal of duplicates and studies that did not meet the eligibility criteria (Fig. 1). Ten studies [19, 24, 26, 27, 42–47]. that met the eligibility criteria were included in the analysis. Studies that were excluded were either conducted in diabetic populations other than GDM or lacked relevant information for calculation of risk estimates. Studies were conducted in the USA, [24, 26, 46]; Brazil, [27, 47]; Spain, [43]; Thailand, [19]; Saudi Arabia, [44]; Turkey, [42] and India [45]. There were six case–control studies [19, 26, 27, 42, 45, 47], three cross sectional studies [43, 44, 46] and one cohort study [24]. Sample sizes ranged from 90 to 4070 and participants’ ages ranged from 14 to 59 years. Studies were published between 2006 and 2015. Definitions used for periodontitis varied widely across studies (Table 1). Seven studies assessed GDM as two or more abnormal values of oral glucose tolerance test (OGTT) [19, 24, 27, 42–44, 47] based on either the O’Sullivan’s [34] or Carpenter and Coustan’s [32] criteria (Table 1). One study [27] defined GDM based on at least one abnormal value of the OGTT [48] and another defined GDM as a positive glucose challenge test [45]. GDM was self-reported in the remaining study [46]. Methodologic quality across case–control and cohort studies was generally high. Six case–control studies and one cohort study were scored either 8 or 7 stars and considered to be of high [19, 24, 26, 27] or medium [42, 47] quality, respectively. One case–control study was noted as having used [45] an inadequate case definition based on the Newcastle Ottawa scale [37], and was considered low in methodologic quality.

**Table 1:** Characteristics of included studies

Author, year (Country)	Study population	Exposure definition	Outcome definition	Risk estimate Confounders	Main conclusion	Study Quality
Chokwiriyachit A et al., [19] (Thailand)	Hospital based, case–control, 2009 to 2010 Age range 14–45, 50 GDM 50 controls	1 site with (PD) ≥5 mm and (CAL) ≥2 mm at the same site.	2 step O’Sullivan’s criteria OGTT screening was performed at 24 to 28, and ≥2 abnormal values was diagnostic of GDM	OR = 7.92 (1.66 to 37.7) FH of DM, pre-pregnancy BMI, and weight gain during pregnancy.	Results suggest association between periodontitis and GDM	High
Ruiz DR et al., [47] (Brazil)	Hospital based, case–control, 2011 161 pregnant women, 80 GDM mean age 33 years., 50 controls mean age 27 years.	Probing on six sites per tooth and periodontal parameters were evaluated, GM, PD, CAL, PI, BI, BOP and MI	2 step Carpenter and Coustan criteria. 2 step OGTT screening was performed at 24 to 28, and ≥2 abnormal values was diagnostic of GDM	(GA, age, FPG, pre-BMI, and HbA_1c_)	Periodontitis was significantly higher in Brazilian diabetic pregnancies (GDM and T1DM) compared to non-diabetic pregnant women	Medium
Xiong, X et al., [26] (USA)	Hospital based, case–control 2007 and 2008, 159 pregnant women, 53 GDM mean age 29.9, 106 controls mean age 27.1	Any site with a PD ≥ 4 mm or a CAL ≥ 4 mm.	2 step Carpenter and Coustan criteria. 2 step OGTT screening was performed at 24 to 28, and ≥2 abnormal values was diagnostic of GDM	2.6 (1.1–6.1) Age, parity, race, marital status, education, income, smoking, alcohol, antibiotics use, family history of DM, income, dental insurance BMI	This study supports the hypothesis of an association between periodontal disease and GD	High
Dasanayake AP et al., [24] (USA)	Hospital based, nested case–control, 262 pregnant women, 22 GDM mean age 28.7, 240 controls mean age 26.6	≥1 pocket with PD > 3 mm	2 step Carpenter and Coustan criteria. 2 step OGTT screening was performed at 24 to 28, and ≥2 abnormal values was diagnostic of GDM	OR = 1.68 (0.52-5.43) (Bivariate analysis) Prior GDM, pre-pregnancy BMI	Periodontal disease did not differ b/n those with GDM and controls	High
Lima E et al., [27] (Brazil)	Hospital based, case–control, 2010 to 2011 360 pregnant women, aged 18–44 mean age 27.2, 90 GDM mean age 32.9, 270 control mean age 25.3	BOP, PD, CAL in 4 sites of all present teeth. ≥4 teeth, with >1 site having PD ≥4 mm was diagnostic	ADA standard for screening and diagnosis of GDM	OR = 0.74 (0.40 to 1.38) Maternal age, marital stability, parity, smoking, alcohol consumption, chronic HPN and BMI	High prevalence of periodontitis was found among cases and controls with no association between periodontitis and GDM	High
Mishra P et al. [45] India	Hospital based, 2009 to 2011 case–control, 90 pregnant women, 30 GDM with mean age 28, 60 control mean age 24	Any site with PD ≥ 4 mm and clinical AL ≥ 3 mm	1-h, 50-g oral glucose challenge test (GCT). If the glucose level was >135 mg/dl (GCT positive)	Generalized: OR = 0.49 (0.07 to 3.52) Localized: OR = 0.54 (0.08 to 3.79)	The results of this study showed that periodontal disease is not significantly associated with GDM	Low
Bagis et al. [42] (Turkey)	Hospital based, 2004 to 2005 case–control, 165 pregnant women, 85 GDM: 80 control, with mean age 28, 60 control mean age 25.85	Assessed using PI; GI; PPD; BOP	2 step Carpenter and Coustan criteria. 2 step OGTT screening was performed at 24 to 28, and ≥2 abnormal values was diagnostic of GDM	NR	Compared to healthy pregnant women, the values GI and BOP were significantly higher for women with GDM	Medium
Bullon P, et al. (2013) (Spain)	Hospital based, cross-sectional, 2013, 188 pregnant women ages 16–44 years., 26GDM, 162 controls	≥2 interproximal sites with CAL ≥6 mm (not on the same tooth) and ≥1 interproximal site with PD ≥5 mm	2 step O’Sullivan’s criteria OGTT screening was performed at 24 to 28, and ≥2 abnormal values was diagnostic of GDM	Periodontitis	Periodontitis in GDM vs. No-GDM (15.5 % vs. 5.6 %; *P* = 0.086) Plague positive OR = 1.012 (1.0 to 1.02). Periodontal disease may be associated with GDM	Not assessed
Habib FA et al. [44] (Saudi)	Hospital based, 250 pregnant women, mean age 32, 100 GDM, 100 pregnant non-GDM and 50 non pregnant women	The Community Periodontal Index of Treatment Needs (CPITN)	2 step Carpenter and Coustan criteria. 2 step OGTT screening was performed at 24 to 28, and ≥2 abnormal values was diagnostic of GDM	NR	GDM; showed significant positive correlation between CPITN scoring	Not assessed
Novak, KF et al. [46] (USA)	Cross-sectional, National Health and Nutrition Examination Survey (NHANES) III sample 4070 pregnant women, age 20 to 59, 88 GDM, 3982 controls	≥1 teeth with ≥1 site with probing pocket depth ≥ 4 mm, clinical attachment loss ≥ 2 mm and bleeding on probing	GDM: Self reported	OR = 2.7 (0.7 to 10.5) Age, presence of sub-gingival calculus, history of smoking, and income	GDM was associated with severe periodontal disease than those without GDM, the association was not statistically significant	Not assessed

*BI* Bleeding Index (BI), *BOP* Bleeding On Probing, *BMI* Body Mass Index, *CAL* Clinical Attachment Level, *FH* Family History, *GDM* Gestational Diabetes Mellitus, *GM* Gingival Margin location, *GI* gingival index, *MI* Tooth mobility Index, *NA* Not Assessed, *PD* Probing Depth, *PI* Plaque Index, *PPD* probing pocket depth

### Synthesis of results

#### Case–control studies

Of the six case–control studies on this topic, 4 studies reported positive associations between periodontal diseases. Chokwiriyachit et al. [19] evaluated women 14 to 44 years in Thailand, and compared 50 cases of GDM with 50 controls. Investigators observed significantly higher prevalence of periodontitis among cases compared to controls (50 % vs.26 %, *P* = 0.02), OR = 3.00 (95 % CI = 1.19 to 7.56). This association remained statistically significant in models controlling for family history of diabetes mellitus, pre-pregnancy BMI, and weight gain during pregnancy (OR = 7.92, 95 % CI: 1.66 to 37.70). However, confidence intervals were wide, probably due to small sample size. Ruiz and colleagues [47] also examined 80 GDM cases and 50 pregnant non-diabetic controls. They found significantly higher prevalence of gingival bleeding among GDM, compared to controls (98.80 % vs. 84 %; *P* < 0.004). The third case–control study, reported by Xiong et al. [26], enrolled 159 women (53 cases and 106 controls). The authors reported significantly higher prevalence of periodontitis in the GDM group (77.4 %) compared to controls (57.5 %), OR = 2.5 (95 % CI: 1.2 to 5.3). The association was minimally changed and remained statistically significant after adjustment for important covariates including family history of DM, income, dental insurance and BMI, (aOR = 2.6, 95 % CI: 1.1 to 6.1) (Table 1). Bagis and colleagues [42] examined 165 pregnant women, 85 with GDM and 80 controls. Compared to healthy pregnant women, the values for gingival bleeding and bleeding on probing were significantly higher for women with GDM compared to controls.

The two remaining case–control studies [27, 45], and one cohort study [24], reported no statistical significant association between women with GDM and control. Esteves-Lima et al. [27] performed full-mouth periodontal examinations in 90 GDM cases and 270 controls to assess periodontitis. Prevalence of periodontitis was 40 % among GDM cases and 46.3 % among controls (*P* = 0.3). Periodontitis was not significantly associated with GDM in multivariable models controlling for maternal age, chronic hypertension and BMI. In a small case–control study conducted in India [45], Mishra and colleagues included 30 cases of GDM as determined by positive glucose challenge test (GCT) when 1-h 50-g oral glucose level was >135 mg/dl, and 60 pregnant controls with glucose levels less than 135 mg/dl on the GCT. Periodontitis was highly prevalent in the study sample but was not different between GDM cases and controls (87 % prevalence of localized or generalized periodontitis in both groups) in either simple comparisons (*P* = 0.3) or in models adjusting for covariates.

In a prospective cohort study, Dasanayake et al. [24] recruited a largely Hispanic cohort of 262 pregnant women at clinical sites in New York City, of whom 22 (8.3)% were diagnosed with GDM. The difference in prevalence of clinical periodontal diseases between women with GDM (50 %) and women without GDM (37.3 %) was not statistically significant (*P* = 0.38).

#### Cross sectional studies

Two of the 3 cross sectional studies included in this review reported no significant statistical association between periodontitis and GDM. Bullon et al. [43] followed 188 pregnant women in Seville, Spain, recruited at 24 – 28 weeks gestation after testing positive on the O’Sullivan screening test and subsequently being referred for confirmatory OGTT. Of these, 26 were diagnosed with GDM. Periodontal disease was observed in four of the 26 women with GDM (15.5 %) as compared to nine of the remaining 162 (5.6 %, *P* = 0.09). Novak KF et al. [46], used data from the third National Health and Nutrition Examination Survey on 4070 subjects. GDM was reported in 88 women. The prevalence of periodontitis was not statistically significantly different between GDM cases (9.0 %) and controls (4.8 %), OR =2.0 (95 % CI: 0.6 to 6.3), or in models adjusting for potential confounders.

In contrast, among 100 women with GDM, 100 pregnant women with no GDM and 50 non-pregnant women in Saudi Arabia [44], Habib et al. compared periodontal disease according to the Community Periodontal Index of Treatment Needs (CPITN-score 1–4) and observed high prevalence of severe periodontal disease (CPITN-score 3–4) that significantly differed (*P* = 0.001) between pregnant women with GDM (37 %) and pregnant women with no GDM (29 %).

### Studies included in the meta-analysis

#### All studies

**Fig. 2:**
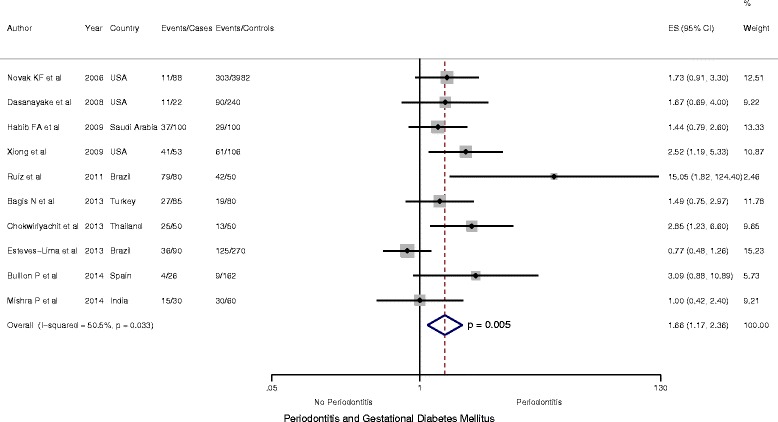
Forest Plot: OR of GDM in Periodontitis: All study studies

Ten studies, including 6 case–control studies [19, 26, 27, 43, 45, 47], 1 cohort study [24] and 3 cross sectional studies [43, 44, 46], enrolling a total of 5724 participants including 624 cases of GDM reported data that were included in the meta-analyses. Random-effects meta-analysis of all ten studies suggested periodontitis to be significantly more common among women with GDM compared with women with no GDM, (OR = 1.66, 95 % CI: 1.17 to 2.36; *P* < 0.05 *I* ^*2*^ = 50.5 %) (Fig. 2).

#### Case–control and cohort studies

**Fig. 3:**
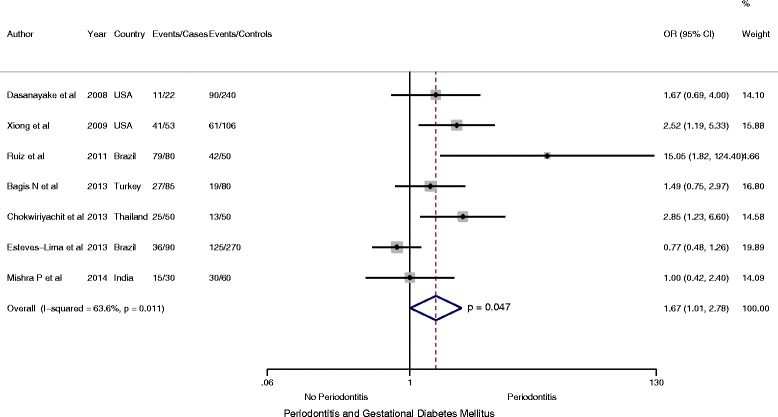
Forest plot: OR of GDM in Periodontitis: Case–control and cohort studies

**Fig. 4:**
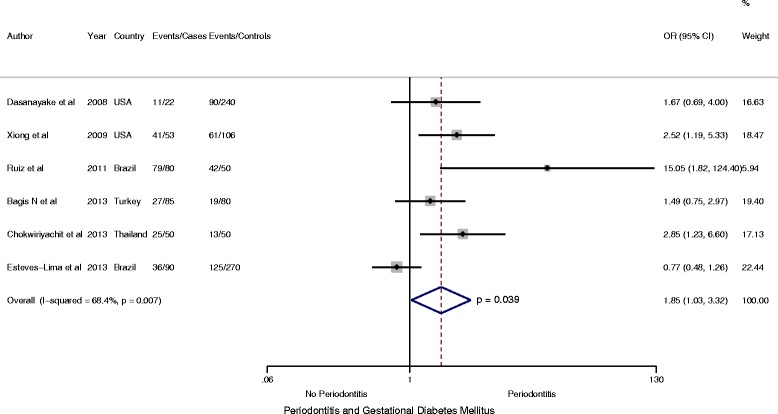
Forest plot: OR of GDM in Periodontitis: Studies of high methodology quality

Similarly, restricted to meta-analysis involving one cohort [24] and six case–control [19, 26, 27, 42, 45, 47] studies, including 1266 participants (410 case and 856 controls), found periodontitis to be a significant risk factor among women with GDM compared with controls, (OR = 1.67, 95 % CI: 1.01 to 2.78; *P* < 0.05, *I* ^2^ = 50.5 %) (Fig. 3). Likewise, when analysis was restricted to 1176 participants including 380 cases of GDM from one cohort and five case–control studies of medium and high methodologic quality, women with GDM were significantly more likely to have periodontitis, (OR = 1.85, 95 % CI: 1.03 to 3.32; *P* < 0.05, *I* ^*2*^ = 68.4.5 %) (Fig. 4).

#### Cross sectional studies

**Fig. 5:**
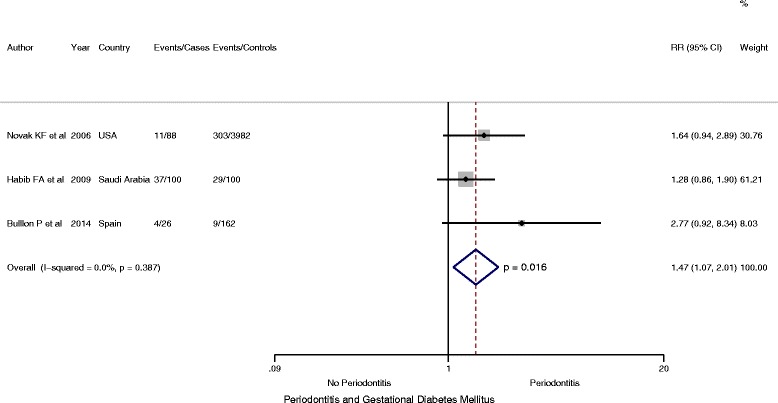
Forest plot: OR of GDM in Periodontitis: Cross sectional studies

Among the three cross-sectional studies that enrolled 4458 participants (214 case and 4244 controls), random-effects meta-analysis yielded a prevalence ratio of 1.47, which was statistically significant (PR = 1.47, 95 % CI: 1.07 to 2.01; *P* =0.016, *I* ^2^ = 68.4 %) (Fig. 5).

#### Studies reporting adjusted estimates

**Fig. 6:**
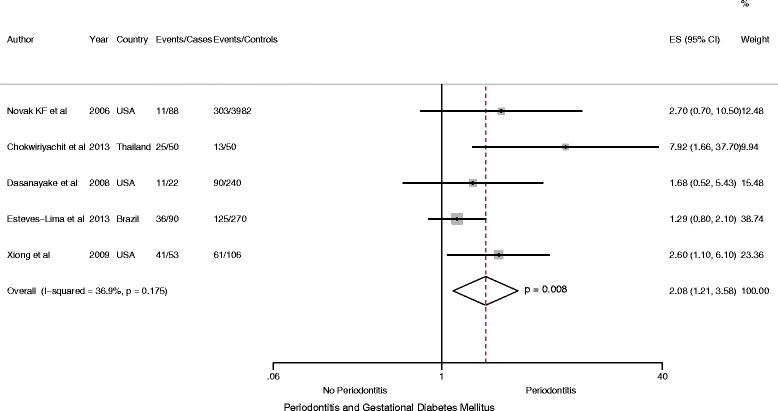
Forest plot: Risk Estimates of GDM in Periodontitis: All Studies with adjusted OR

**Fig. 7:**
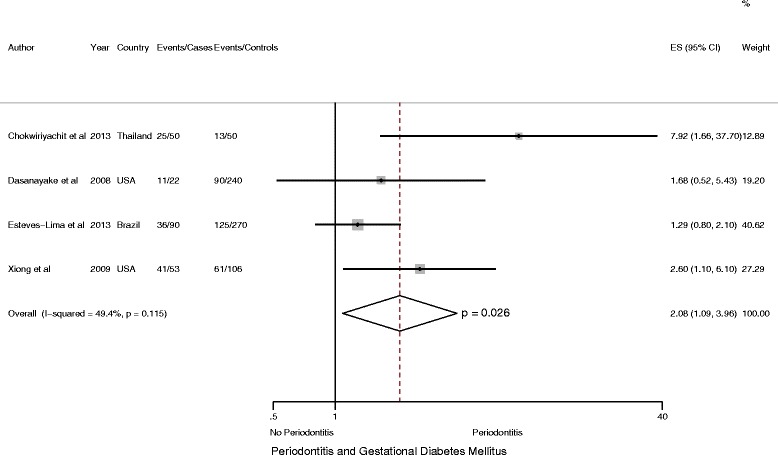
Forest plot: Risk Estimates of GDM in Periodontitis: Case–control studies with adjusted OR

In order to address the potential impact of confounding, we performed separate analyses limited to studies reporting adjusted estimates. In meta-analysis including five studies (4951 participants including 124 cases) that reported information on adjusted effect estimates [19, 24, 26, 27, 46] a pooled adjusted OR of 2.08 (95 % CI: 1.21 to 3.58, *P* = 0.009; *I* ^*2*^ = 36.9 %) was estimated (Fig. 6). Similarly, when consideration was restricted to all the four case–control studies (881 participant including 113 case), that reported adjusted OR, a two-fold increased odds of GDM was observed comparing women with periodontitis to those without (adjusted OR = 2.08, 95 % CI: 1.09 to 3.96; *P* < 0.026; *I* ^2^ = 49.4 %), (Fig. 7).

**Fig. 8:**
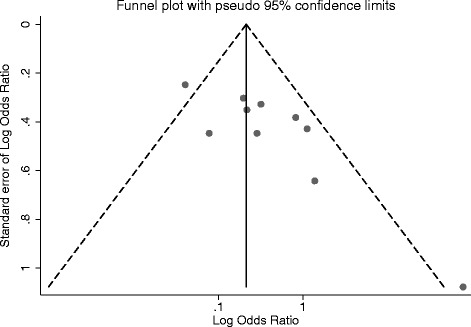
Publication bias

Visual inspection of the funnel plot shows plot symmetry and the Egger test for the degree of publication bias was not statistically significant (*P* = 0.247, Fig. 8).

## Discussion

Results of this meta-analysis suggest that periodontitis is associated with the development of GDM. The association was robust to study design, being observed in overall analysis including all studies and in studies restricted by study design. The results were unchanged when confounding and methodologic quality were taken into consideration; studies reporting adjusted estimates as well as those case–control studies with medium and high methodologic quality both yielded estimates further from the null than the overall estimate. Evidence continues to accumulate that periodontal disease is a risk factor for GDM and other pregnancy outcomes. GDM is a disorder of multiple risk factors including obesity [6] family history, previous history of GDM, advanced maternal age and polycystic ovarian syndrome [7], the combination of which may act synergistically to cause GDM. We observed a more than 2-fold increased risk between GDM and periodontitis (aOR = 2.08) in studies that adjusted for such described risk factors for GDM. This finding is consistent with those of previous reviews on this topic [28, 49].

The mechanism by which periodontal disease causes GDM is not yet fully elucidated [50]. Evidence of elevated levels of inflammatory mediators such as leucocytes and C reactive proteins in systemic circulation of women with GDM suggests the possibility of a nidus of chronic infection in the body that may play a vital role [11–13]. It is believed that that periodontitis is capable of inducing local and host immune responses causing both transient bacteremia and the release of inflammatory markers such as interleukins and tumor necrosis factors, which then can act multiplicatively to block the effect and action of insulin, or act via the destruction of pancreatic beta cells to prevent its production. This process consequently leads to insulin resistance and impairment of glucose metabolism and, if not reversed, to GDM [18, 19, 51].

Research in this area is challenged by variability in the diagnosis of periodontitis and difficulty in knowing precisely what to measure, as well as the different clinical criteria and definitions used to assess prevalence of GDM across studies. Nevertheless, findings of this study suggest that periodontitis significantly increases the risk of GDM, which is consistent with previous studies [28, 49].

Among the strengths of our study is the relatively large sample size resulting from inclusion of data from ten studies on periodontitis and GDM. Furthermore, most of these studies were determined to be at low risk of bias based on the NOS scale of bias tool, supporting the validity of the meta-analytic results. Because of the available sample size, we were able to perform subgroup analyses, including one using studies that only reported adjusted estimates, which helps accounts for the possibility of confounding affecting our findings. Additionally, we assessed the risk of publication bias by visual inspection of funnel plot for symmetry and Egger test, both of which suggested lack of publication bias (Fig. 8).

This review is not without limitations. First, the clinical criteria for assessing periodontal disease are non-uniform and tend to introduce non-differential misclassification of periodontitis, with a potential effect of driving the results of the study towards the null - this may explain the lack of significant association found in some of the individual included studies. Meta-analysis is an effective approach for evaluating exposure effects when individual studies may be underpowered to detect clinically important effects due to limited sample size or non-differential misclassification.

Second, confounding is always a concern for observational studies. Although we performed meta-analysis of both unadjusted and adjusted estimates and did not observe substantial differences between the two, some included studies did not report covariates included in adjusted models. Moreover, unmeasured confounders may exist and could lead to over estimation of the results. For instance, a genetic link between periodontics and GDM may exist [52], as some studies have postulated a possible existence of genetic polymorphism between inflammatory cytokines such as tumor necrosis factor alpha, interleukin-1 and interleukin-6, insulin resistance and periodontitis [53, 54] such that their derangement may concurrently cause periodontitis and GDM. Future studies should consider all potential confounders, including those that have not previously been considered.

Third, GDM was evaluated based on different criteria of at least 2 abnormal values of OGTT with different cut off values [32, 34] or based on at least one abnormal value of OGTT [51]. Because GDM has serious complications and can cause adverse pregnancy outcomes if not detected early and promptly treated, newer guidelines recommend the screening and diagnosis of GDM based on one abnormal value of OGTT in an attempt to identify all possible GDM candidates. This method is prone to overestimate GDM by 18 % [51] and has the potential to non-differentially misclassify GDM. We restricted inclusion to studies utilizing standard GDM diagnostic approaches, and despite potential non-differential misclassification of GDM status observed a statistically significant two-fold increased odds of disease with periodontitis; however, accurate and standard procedures for diagnosis of GDM are important in order to avoid biases in future research.

Fourth, some important issues arise related to study design. For example, temporal ordering is a significant challenge to studies in this area and is poorly addressed by retrospective designs, or even prospective studies that incompletely capture information on timing. Periodontal disease is a chronic process, and it is unknown how long it takes to affect carbohydrate metabolism or impact other physiological processes that may influence GDM risk. However, pregnancy has duration of approximately 9 months, plus a six-week puerperal period, and whether this period allows sufficient time to observe the effect of periodontitis on GDM remains unclear. Notwithstanding the potential bidirectional effect of periodontitis and GDM [46], and the fact that GDM occurs over a short duration, the likelihood that GDM causes severe periodontal destruction is relatively less than the reverse being the case.

The use of hospital controls in the case–control studies included in this review makes them particularly susceptible to selection bias, which could affect the overall risk estimate. Moreover, because of retrospective reporting, case–control studies are also subject to recall bias and errors, the presence of which can lead to under or overestimation of the exposure and a possible bias to the risk estimate. Among the case–control studies included in this review periodontitis was assessed by trained clinicians, which reduces the likelihood of such misclassification. Furthermore, the cross sectional design used in some of the included studies makes it impossible to assess the temporality between periodontal disease and GDM. To minimize the potential influence of variation in the methods of assessing periodontitis and GDM as well as differences in study designs on the risk estimates, all analyses were conducted using random effects model. However, similar results were found when we repeated the analysis using fixed effects model (data not shown).

Finally, although we observed funnel plot symmetry and the Egger test was not statistically significant, publication bias and its associative selective outcome reporting of studies with positive findings cannot be completely ruled out. If null studies are underrepresented in the available data, it would constitute a potential bias in our effect estimates.

However, findings of our meta-analysis of ten studies add to the growing evidence supporting an association between periodontitis and the risk of GDM. There has been substantial recent attention to potential relations of oral infection and reproductive outcomes; as a result, our analysis provides an update with additional statistical power to a recent review that found the evidence for this association to be inconclusive [30].

Our results suggest that periodontitis potentially increases the risk for GDM approximately 2-fold. Periodontitis is a treatable condition; research aimed at determining how periodontitis influences risk of GDM is an important first step to guide public health prevention programs and treatment strategies to minimize complications. Lack of prospective cohort studies, the clinical variability of definitions and methods to accurately classify periodontitis and GDM, are major limitations in the field. We hope these findings stimulate interest in this area and lead to further research to address remaining questions and clarify the role of periodontitis in GDM.

## Conclusions

The results of this systematic review and meta-analysis suggests periodontitis to be associated with the development of GDM; however, the establishment of a clear causal relationship awaits further research in terms of robust prospective study designs, consideration of issues of temporal ordering, and appropriate tools for, and consistent definitions of, determination of periodontal disease. This strong evidence of association has important implications for public health, especially for pregnant women, and should activate new intervention strategies for professionals in dental medicine, and obstetrics and gynecology.

## Abbreviations

BI: Bleeding index
BMI: Body mass index
BOP: Bleeding on probing
CAL: Clinical attachment level
FH: Family history
GDM: Gestational diabetes mellitus
GI: Gingival index
GM: Gingival margin location
MA: Massachusetts
MI: Tooth mobility index
NA: Not assessed
OR: Odds ratio
PD: Probing depth
PI: Plaque index
PPD: Probing pocket depth
